# Small GTPase Rab40c Associates with Lipid Droplets and Modulates the Biogenesis of Lipid Droplets

**DOI:** 10.1371/journal.pone.0063213

**Published:** 2013-04-30

**Authors:** Ran Tan, Weijie Wang, Shicong Wang, Zhen Wang, Lixiang Sun, Wei He, Rong Fan, Yunhe Zhou, Xiaohui Xu, Wanjin Hong, Tuanlao Wang

**Affiliations:** 1 School of Pharmaceutical Sciences, State Key Laboratory of Cellular Stress Biology, Xiamen University, Xiamen, Fujian, China; 2 Institute of Molecular and Cell Biology, A*STAR (Agency for Science, Technology and Research), Singapore, Singapore; Fundação Oswaldo Cruz, Brazil

## Abstract

The subcellular location and cell biological function of small GTPase Rab40c in mammalian cells have not been investigated in detail. In this study, we demonstrated that the exogenously expressed GFP-Rab40c associates with lipid droplets marked by neutral lipid specific dye Oil red or Nile red, but not with the Golgi or endosomal markers. Further examination demonstrated that Rab40c is also associated with ERGIC-53 containing structures, especially under the serum starvation condition. Rab40c is increasingly recruited to the surface of lipid droplets during lipid droplets formation and maturation in HepG2 cells. Rab40c knockdown moderately decreases the size of lipid droplets, suggesting that Rab40c is involved in the biogenesis of lipid droplets. Stimulation for adipocyte differentiation increases the expression of Rab40c in 3T3-L1 cells. Rab40c interacts with TIP47, and is appositionally associated with TIP47-labeled lipid droplets. In addition, over-expression of Rab40c causes the clustering of lipid droplets independent of its GTPase activity, but completely dependent of the intact SOCS box domain of Rab40c. In addition, Rab40c displayed self-interaction as well as interaction with TIP47 and the SOCS box is essential for its ability to induce clustering of lipid droplets. Our results suggest that Rab40c is a novel Rab protein associated with lipid droplets, and is likely involved in modulating the biogenesis of lipid droplets.

## Introduction

Lipid droplets (LDs, also referred to as adiposome) are the unique organelle for the storage of neutral lipids, mostly triacylglycerols (TG) and cholesterol esters. Lipid droplet has a lipid core surrounded by a monolayer of phospholipids membrane, and is produced by many types of cells in animals, plants and even microorganisms (comprehensively reviewed in [1]–[3]). Recent studies revealed that LD is not a static structure for the storage of lipid, but a dynamic organelle to maintain lipid homeostasis, generate energy and regulate cell signaling [4]–[8]. Excessive lipids in LDs can be metabolized through lipolysis and oxidation to generate ATP, and also be transported to other organelle as the components of membrane biogenesis. Dysfunction of LDs is related to many metabolic diseases, such as obesity, diabetes and arteriosclerosis (reviewed in [9], [10]).

It is widely accepted that LDs are primarily derived from endoplasmic reticulum (ER) membrane [11]–[13], althoughthe plasma membrane, endosomes and mitochondria may also contribute to the biogenesis of LDs [14]–[17]. However, the regulatory mechanisms for the biogenesis of LDs remain elusive. Characterization of proteins associated with LDs will be helpful for understanding the biogenesis and dynamic interaction of LDs with other cellular structures.

The best characterized LDs surface proteins are the PAT proteins [perilipin, ADRP (adipocyte differentiation related protein or adipophilin) and TIP47 (tail-interacting protein 47 KDa)], which are classified by harboring the perilipin amino-terminal (PAT) domain within the N-terminal portion of these proteins. PAT proteins are crucial for LDs function and biogenesis [18]–[22]. Investigations revealed that perilipin, ADRP and TIP47 are present in different stages during adipocyte differentiation [23], but it is not clear how these PAT proteins are regulated to target to LDs. In addition to the PAT proteins and enzymes for lipid metabolism, LDs contain membrane trafficking machineries, such as SNARE and Rab proteins, which may regulate the biogenesis, fusion, fission and movement of LDs [4], [24], [25].

Proteomics studies demonstrated that many Rab proteins associate with lipid droplets [26]–[28]. Rab small GTPases are the key regulators in membrane trafficking, probably also modulating biogenesis of LDs and their interaction with other organelle. Rab18 is the best characterized Rab protein associated with LDs. Rab18 localizes to LDs reciprocally with ADRP, and induces the apposition of LDs to the ER [29]–[30]. Lipolytic stimulation in adipocytes increases the association of Rab18 to LDs [31]. These results indicate that Rab18 is involved in the biogenesis of LDs, however, the underlying mechanisms needs further investigations.

Rab40c is a homolog of xRab40 in Xenopus, which was shown to interact with culin5 and elongin B to form a complex, serving as E3 ligase and regulating noncanonical wnt pathway [32]. Mammalian Rab40c was suggested to play a role in vesicle transport in oligodendrocytes [33]. In this study, we presented data to demonstrate that Rab40c associates with LDs and is likely involved in their biogenesis.

## Materials and Methods

### Antibodies

The rabbit polyclonal antibody against Rab40c was generated by Genemed Synthesis (San Antonio, TX) using the peptide sequence (LMRHGMEKIWRPNRVFS) as the antigen. The monoclonal antibodies (mAbs) against GM130 and EEA1 were from BD (BD Biosciences, PaloAlto, CA), mAbs against rat Lamp2 was obtained from the Developmental Studies Hybridoma Bank maintained by the University of Iowa (Department of Biological Science, Iowa City, IA). mAb against Myc-tag (9E10) and mAb against Flag tag were obtained from American Type Culture Collection (ATCC, Manassas, VA). mAb against lysobisphosphatidicacid (LBPA) was a generous gift from Dr Jean Gruenberg(University of Geneva, Switzerland). mAb against KDEL receptor was from BD (BD Biosciences, PaloAlto, CA). Rabbit polyclonal antibody against ADRP was from SANTA CRUZ (Delaware Avenue, CA). Rabbit polyclonal anti-TIP47 antibody was purchased from AnaSpec (Fremont, CA). Rabbit mAb against Perilin was from Cell Signaling (Danvers, MA). mAb against GFP was from Clontech (Palo Alto, CA,USA). mAb against β–tubulin was from Sigma (St. Louis, MO). HRP-conjugated secondary antibodies were purchased from Pierce (Rockford, IL). Texas red-conjugated secondary antibodies were from Jackson ImmunoResearch (West Grove, PA).

### Expression Constructs

The coding regions of human Rab40a, Rab40b and Rab40c were retrieved from the cDNA library of Human fetal brain (BD Clontech) by PCR with primer1(5-ATGAATTCAATGAGCGCCCCG GGCAGCCCCG-3) and primer 2 (5-ATATGTCGACTTAAGAAATTTTGCAGCTGTT-3) for Rab40a, primer 3(5-CGGAATTCAATGAGCGCCCTGGGCAGCCCGGT-3) and primer 2 for Rab40b, and primer 4(5-CGGAATTCGGGCTCGCAGGGCAGTCCGGTG-3) and primer 5 (5-ATATGTCGACCTAGGAGATCTTGCAGTTACTCCG-3) for Rab40c, respectively, and then cloned into EcoR I/Sal I sites of pEGFP-C1 vector (BD Clontech) to generate constructs for expression of GFP-Rab40a, Rab40b and Rab40c with N-terminal fusion of GFP to preserve the C-terminal prenylation signal. The mutants of Rab40cG28N, Rab40cG28T, Rab40CQ73L and SOCS mutant Rab40c LPLP(212-215)AAAA were generated by PCR directed mutagenesis approach. pCIneo-Rab40c was constructed by sub-cloning the coding region of Rab40c into pCIneo vector (Promega Biotech, Beijing, China). The truncated forms GFP-Rab40c(11-281) and GFP-Rab40c(1-243) were also constructed by PCR technology. All constructs were confirmed by DNA sequencing. The coding regions for Rab40b and Rab40c were sub-cloned into pGEX-4T-1 vector (Amersham Biosciences, Arlington Heights,IL) to generate the constructs for expression of GST-Rab40b and GST-Rab40c. GFP-ERGIC-53 is a gift from Dr. Lei Lu (Nanyang Technological University, Singapore). pEF.flag-Rab18 is a gift from Dr. Fukuda Mitsunori (Brain Science Institute, RIKEN, Saitama, Japan).

### Tissue Expression Assessed by cDNA Panel

Human multiple tissues cDNA panels (BD Biosciences, Palo Alto, CA,USA) were used for PCR-based analysis to assess the expression levels of the transcripts for Rab40a, Rab40b and Rab40c using the primers described above. Primer 8 (5-TGAAGGTCGGAGTCAACGGATTTGGT-3) and primer9 (5-CATGTGGGCCATGAGGTCCACCAC-3) were used to amplify cDNA fragment of G3PDH as a control. The PCR products were resolved by agarose gel electrophoresis.

### Cell Culture, Plasmids Transfection and Adipocyte Differentiation Induction

NRK cells, HepG2 cells, Hela cells and MCF7 cells were from ATCC and grown in DMEM media supplemented with 10% fetal bovine serum (Gibco, Ann Arbor, MI) in a 5% CO2 incubator at 37°C. 3T3-L1 cells were grown in DMEM medium (4.5 g/liter) supplemented with 10% newborn calf serum. Transient transfection of plasmids was conducted using either Lipofectamine 2000 reagents (Invitrogen,Gaithersburg, MD, USA) or TurboFect in vitro transfection reagent (Thermo, Massachusetts, USA) according to the manufacturer’s protocol. When inducing lipid droplets formation in HepG2 cells, 100 µg/ml oleic acid (pre-complexed with 0.1% fatty-free BSA in 0.1 M Tris·Cl, pH8.0) was added to the media. For serum starvation, cells were grown in DMEM medium (4.5 g/liter) without FBS.

Adipocyte differentiation was induced as described previously [34]. Briefly, 3T3-L1 fibroblasts cells in 100% confluence were initiated to induce adipocyte differentiation in differentiation media (DMEM containing 10% newborn calf serum, 0.05 mM isobutyl methylxanthine, 1 µM dexamethasone and 10 mg/ml insulin). The differentiation media is replaced with DMEM (containing 10% newborn calf serum and 1 mg/ml insulin) after 3 days of induction. Cells were finally maintained in normal DMEM (containing 10% newborn calf serum) after 6 days of induction.

### Immunofluorescence Microscopy

Immunofluorescence microscopy experiments were performed as described [35]. Briefly, cells grown on coverglasses were washed with PBSCM (PBS containing 1 mM CaCl_2_ and 1 mM MgCl_2_) and then fixed with 3% paraformaldehyde in PBSCM at 4°C for 30 min. After washing with PBSCM supplemented with 50 mM NH_4_Cl, cells were permeabilized with 0.1% saponin (Sigma, St. Louis, MO, USA) in PBSCM for 15 min at room temperature, and were subjected for immuno-staining using the antibodies indicated, followed by Texas red-conjugated secondary antibodies. GFP-proteins were were viewed directly under GFP channel in microscope. The immuno-labeled cells were analysed with Carl Zeiss LSM5 EXITER laser scanning confocal microscope (Zeiss, Jena, Germany).

### Lipid Droplets Fractionation

[]Lipid droplets fractionation was performed as described [30], with some modifications. Briefly, HepG2 cells from seven 10 cm-dishes, induced with oleic acid, were harvested by scraping with disruption buffer (25 mM Tris/HCl, pH 7.4, 100 mM KCl 1 mM EDTA and 5 mM EGTA, protease inhibitor cocktail (Roche)). The cells (2.0 ml) were disrupted by five passes through a 27 gauge needle, and then spun at 1000×g for 10 min at 4°C. The supernatant, adjusted to 0.33 M sucrose in a final volume of 3.0 ml, was overlaid with 3.0 ml of 0.25 M sucrose, 0.125 M sucrose in disruption buffer and 3.0 ml of disruption buffer, followed by centrifugation at 150,000×g for 2 h in a Beckman SW41 rotor. Eight fractions (1.5 ml each) were collected from the top of the tube, and assessed for western-blotting analysis.

### GST-pulldown Assay and Western–blot Experiments

GST-pulldown assay was performed as described previously [35].Briefly, MCF7 cells were used to achieve high transfection efficiency when detecting protein level, and were transfected with Rab40c expression constructs to generate cell lysates containing the proteins indicated in the text, and the GST-fusion proteins immobilized onto the GST-Sepharose-4B resin were used to bind the interacting proteins in the cell lysates.

For western-blotting experiments, tissue lysates or the samples from GST-pull-down assay were resolved by SDS-PAGE and transferred to nitrocellulose filter. The filter was blocked with 5% milk in PBS and then incubated with primary antibody for 1 h at room temperature, followed by the incubation with HRP-conjugated secondary antibody. The blots were detected using ECL system (Pierce, Rockford, IL, USA).

### Small Hairpin RNA Interfence

pSuper vector mediated shRNA was carried out by constructing Rab40c targeting sequence (5′-AAGGAGATCGATGAGCATGCA-3′) into pSuper.GFP.neo vector (Oligoengine) to generate pSuper.shRNA-Rab40c. The constructs expressing shRNA were transfected into the indicated cells to inhibit the expression of Rab40c, and the knockdown effects were examined 72 hours after transfection. The sequence (5′GATGCAACCACCCACGAAT3’) was used for control shRNA.

### Statistical Analysis

Mean size of lipid droplets, from 50 cells of transfected cells, was estimated using the software ImageJ 1.32 (NIH) under confocal microscope. The size differences between groups were examined with *q test* (Newman-Keuls test), and p<0.05 was considered statistically significant.

## Results

### Rab40c has Similar Tissue Expression Pattern as Rab40a and Rab40b but Exhinits Distinct Subcellular Localization

**Figure 1:**
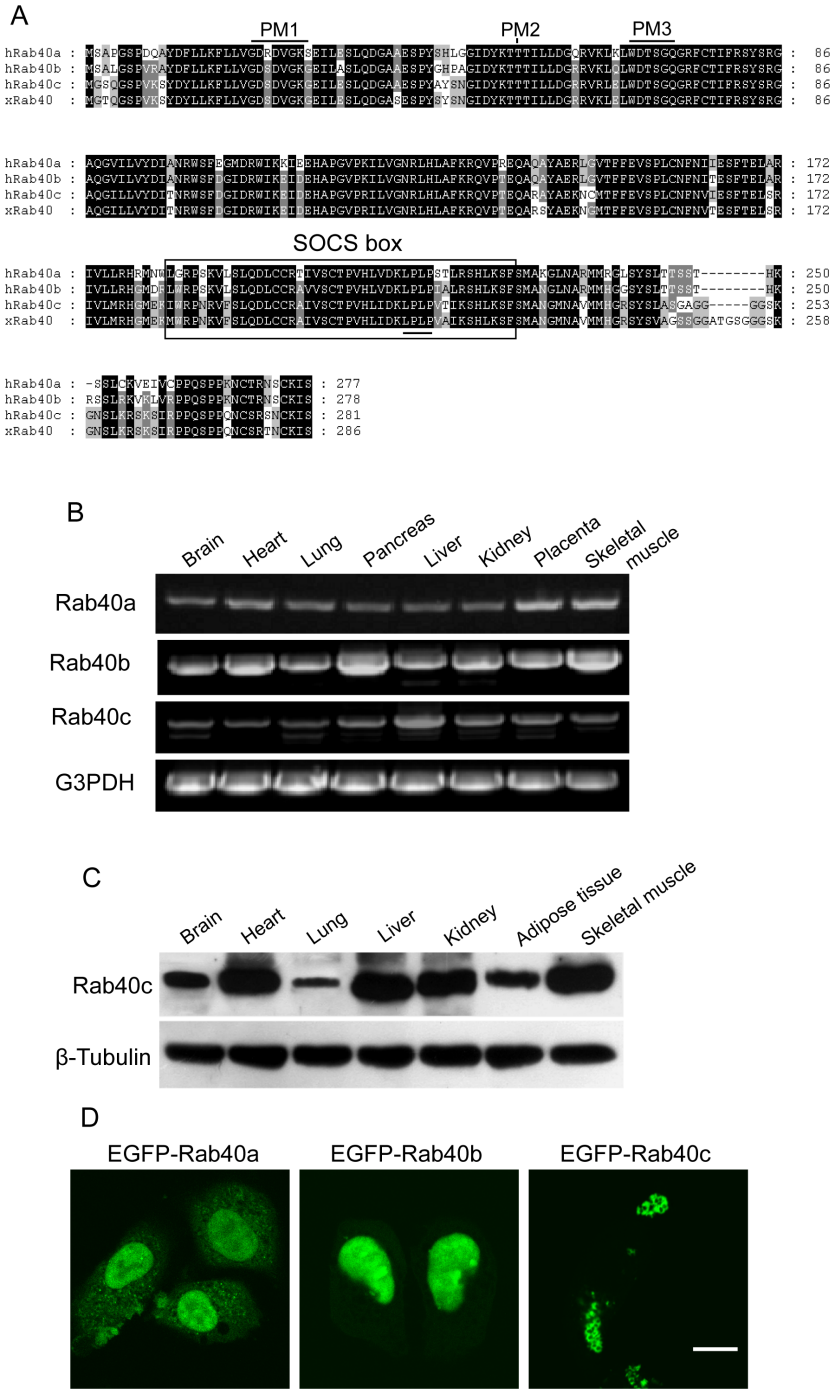
The expression of Rab40c. A. sequence alignment showed that Rab40 family proteins possess conserve phosphate/Mg2+ (PM) binding motifs and SOCS box (the key residues for proper function of SOCS box are underlined), and hRab40c is likely the homolog of xRab40. B. PCR based examination of the transcript levelsof 3 Rab40 members. C. Rab40c is expressed in different tissues as examined by western-blot using Rab40c antibody. D. NRK cells were transfected with GFP-Rab40a, GFP-Rab40b or GFP-Rab40c, respectively. Immuno-fluorescence microscopy analysis showed different localization of Rab40 family proteins. Bar, 20 µm.

XRab40 regulates *Xenopus* gastrulation, and may carry out it biological functions through interaction with Cullin5-ElonginB to serve as E3 ligase [32]. Thus we are interested in the cell biological function of Rab40 proteins in mammalian cells. Interestingly, there are 3 members of Rab40 referred to as Rab40a, Rab40b and Rab40c in mammalian cells. Sequence alignment showed all members possess a unique SOCS box at the C-terminal region, which is absent in other Rab proteins. Rab40c seems to be the human homolog of XRab40c with 93% amino-acid identity (Figure 1A). Like other Rab proteins, Rab40 family proteins have some conserved motifs for GTPase activity and guanine nucleotide binding, as indicated in Figure 1A, Rab40 proteins has PM1, PM2 and PM3 motifs for phosphate/Mg2+ binding, but lacking the guanine-based binding motifs G1, G2 and G3, which are conserved in other Rab proteins [36]. In addition, the PM1 motif in Rab40b or Rab40c is GxxxGKG, normally GxxxGKS/T in other Rab proteins. This analysis suggests Rab40 family proteins may have lower GTPase activity.

PCR based examination of the transcript levels of Rab40 members demonstrated that they are expressed ubiquitously in different human tissues (Figure 1B), suggesting all 3 members are involved in general biological processes in mammals. We further detected the protein levels of Rab40c in rat tissues, and found Rab40c is distributed in different tissues, consistent with the expression of its transcript in human tissues (Figure 1C).

In order to study the cell biological function, we generated GFP-tagged Rab40a, Rab40b and Rab40c, and examined their sub-cellular location using immuno-fluorescence microscopy. Surprisingly, these 3 members have different subcellular locations. GFP-Rab40a and GFP-Rab40b are generally distributed in the cytoplasmnucleus. Interestingly, GFP-Rab40c is mostly associated with vesiclar structures (Figure 1D), suggesting Rab40c may be involved in a cellular events different from Rab40a and Rab40b.

### Rab40C Associates with LDs

**Figure 2:**
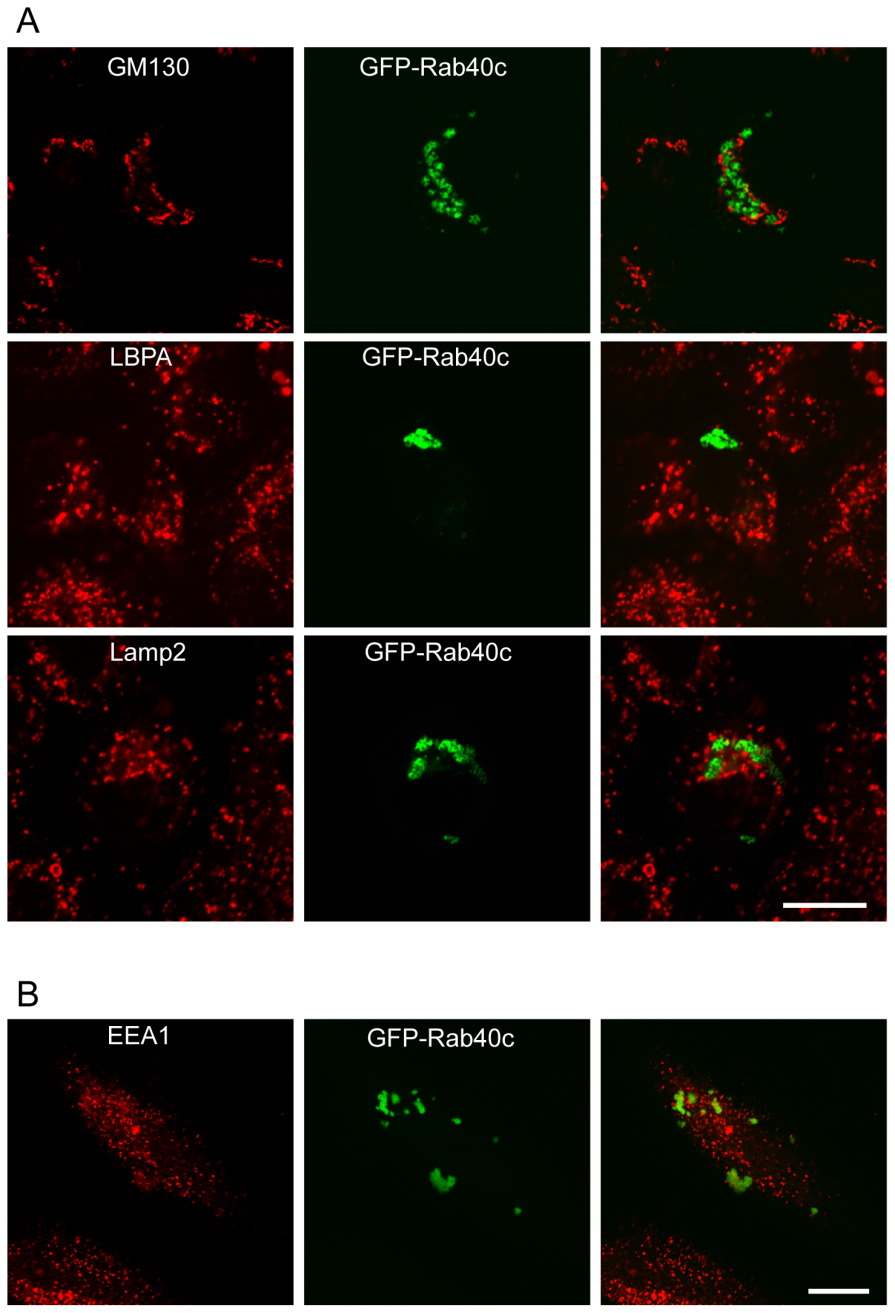
Rab40c is not associated with endocytic compartments and the Golgi apparatus. A. NRK cells were transfected with GFP-Rab40c, and then immuno-labeled with the indicated antibodies. Immuno-fluorescence microscopy showed that GFP-Rab40c is not co-localized with Golgi marker GM130, late endosomal/lysomomal markers LBPA and Lamp2 in NRK cells. B. GFP-Rab40c is not co-localized with early endosomal marker EEA1 in Hela cells. Bar, 20 µm.

To investigate the properties of the GFP-Rab40c-associated vesicles, we performed co-localization experiments with markers for different organelles using immuno-fluorescence microscopy. As NRK cells give a better vesicular structure of Rab40c in our experiments, we examined the sub-cellular localization of Rab40c in NRK cells. It was observed that GFP-Rab40c did not co-localize with Golgi marker GM130, late endosomal/lysomomal markers LBPA and Lamp2 in NRK cells (Figure 2A). EEA1 antibody, not labeling early endosomes in NRK cells as the antibody reacts with only human protein, was used to examine whether Rab40c co-localizes with the early endosomes in Hela cells, and the results demonstrated that Rab40c does not co-localize with EEA1 either (Figure 2B). These results indicate that Rab40c is not associated with endocytic compartments or the Golgi apparatus.

**Figure 3:**
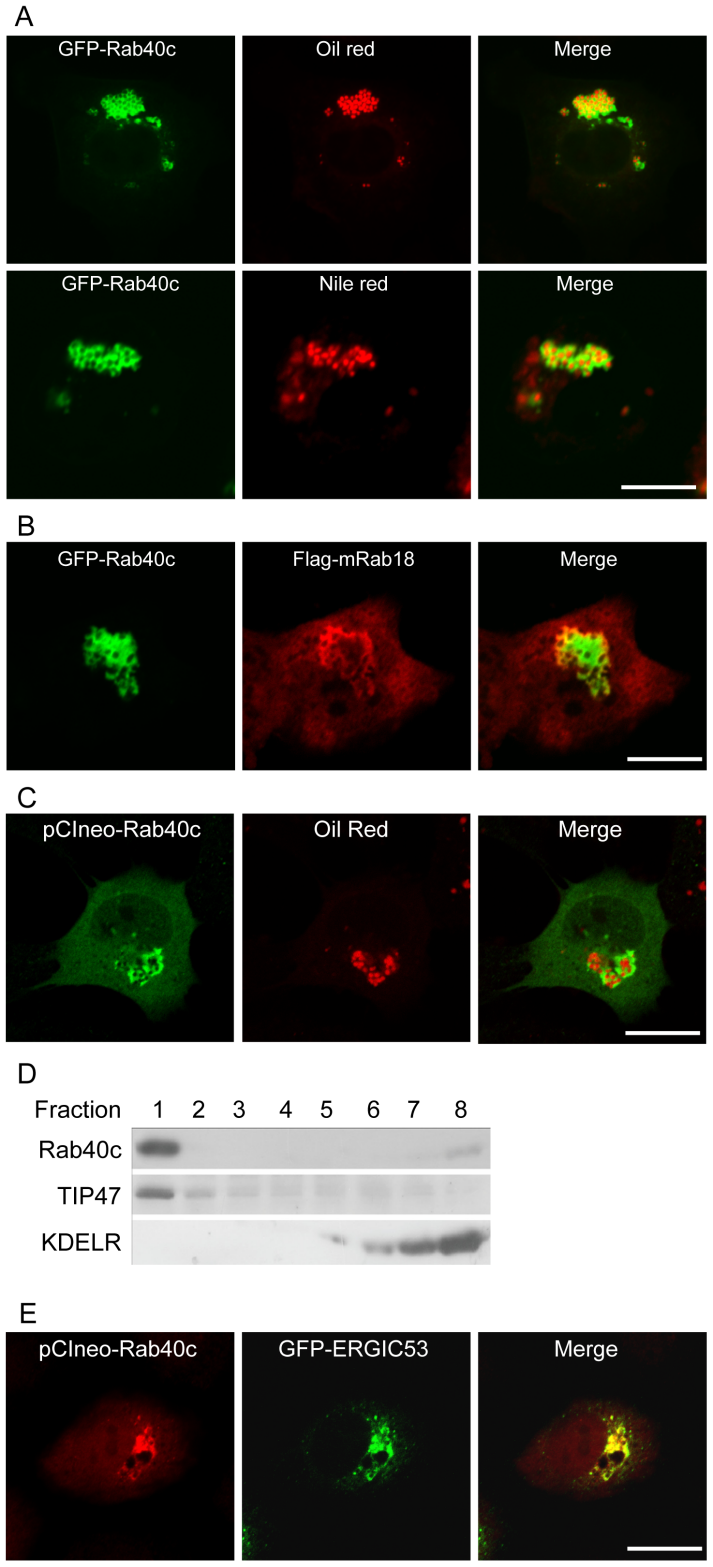
Rab40c is present in lipid droplets and structures marked by ERGIC-53. A. NRK cells were transfected with GFP-Rab40c and labeled with Oil red or Nile red, then processed for immuno-fluorescence microscopy. The results showed that GFP-Rab40c is associated with LDs. B. NRK cells were co-transfected with GFP-Rab40c and Flag-Rab18, and processed for immuno-fluorescence microscopy, Rab40c is revealed by GFP and Rab18 is revealed by labeling with antibody against Flag tag. The results showed that GFP-Rab40c co-localizes partially with flag-Rab18. C. NRK cells were transfected with pCIneo-Rab40c and labeled with Oil red, and immuno-stained by antibody against Rab40c, showing that non-tagged Rab40c is also associated with LDs. D. Western blotting analysis of sucrose density-gradient fractions from HepG2. An equal volume from each fraction was loaded. Both TIP47 and Rab40c were enriched in the top floating LDs-containing fraction (lane 1). The ER-Golgi recycling protein KDEL receptor was detected in the bottom fractions. E. NRK cells were co-transfected with pCIneo-Rab40c and GFP-ERGIC-53, and immuno-stained by antibody against Rab40c. Rab40c is also associated with structures marked by ERGIC-53. Bar, 20 µm.

As GFP-Rab40c is not associated with most of the membrane trafficking compartments, further examinations were conducted to focus on other organelles, such as mitochondria and LDs. Interestingly, GFP-Rab40c was seen to be present on the surface of LDs marked by Oil-red or Nile red in NRK cells (Figure 3A), but was not associated with mitochondria marked by MTOC2 (data not shown). As Rab18 is a well characterized Rab protein associated with LDs, we then examined the co-localization of Rab40c with Rab18, and found that GFP-Rab40c co-localizes with Flag-Rab18 to some extent, but in some vesicles, having high levels of Rab40c seems to have less amount of Rab18 (Figure 3B), suggesting that Rab40c and Rab18 may associate with the distinct populations of LDs or they may regulate LDs association of each other in a negative way. To further confirm the association of Rab40c with LDs, peptide antibody against Rab40c was used to examine the location of endogenous Rab40c, but the antibody is not suitable for immuno-staning of the endogenous Rab40c, likely due to low levels of expression. Thus we generated the expression construct pCIneo-Rab40c to express the wildtype of untagged Rab40c and examined its localization using the Rab40c antibody. The data revealed that exogenous wildtype of Rab40c is also associated with LDs (Figure 3C).

To further confirm Rab40c association with LDs, we purified the LDs fraction from HepG2 cells induced with oleic acid, western-blot analysis of subcellular fractions demonstrated that Rab40c is highly enriched in the top floating fraction, which contains the LDs fractions marked by TIP47 (Figure 3D), although a fraction of Rab40c is also co-fractionated with ER-Golgi resident recycling protein KDEL receptor. Taken together, we concluded that Rab40 is a novel Rab protein associated with LDs.

Although Rab40c does not colocalize with most of trafficking compartments, some Rab40c was observed to associate with ER-Golgi intermediate compartments. when closely examined by co-expressing pCIneo-Rab40c with GFP-ERGIC-53 (Figure 3E), indicating Rab40c may dynamically associate with different membrane populations, which is consistent with the fractionation results.

### Rab40c is Likely Involved in the Biogenesis of LDs

**Figure 4:**
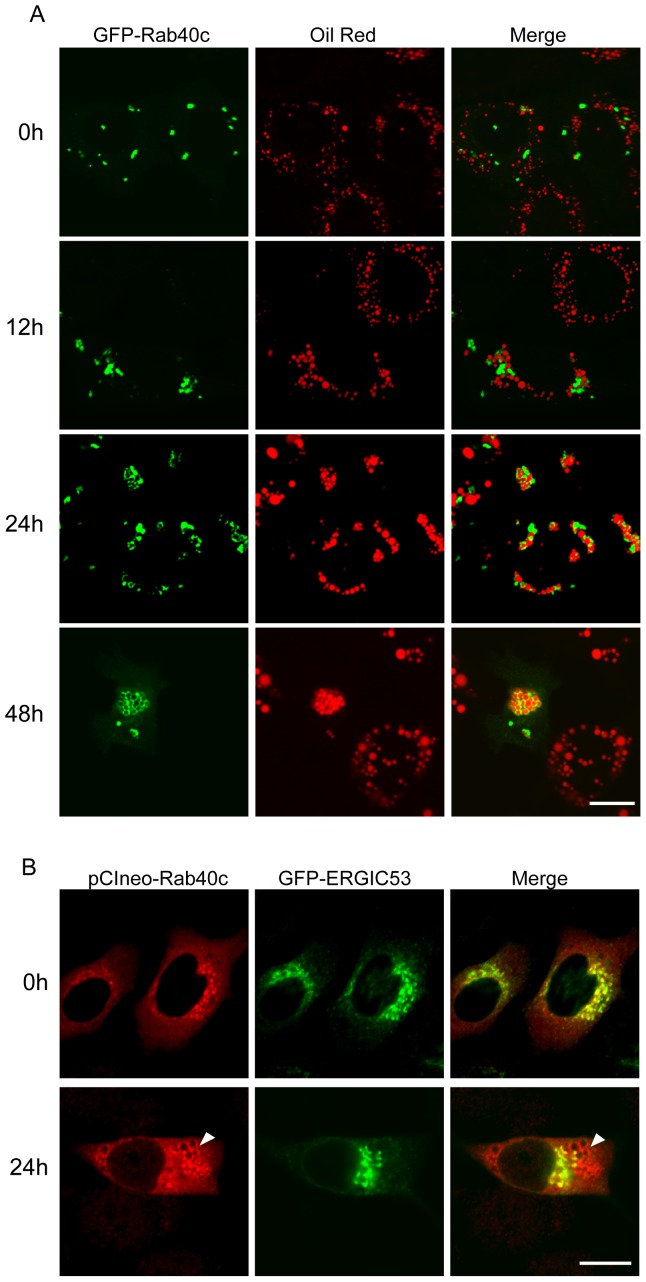
Rab40c is recruited to the LDs. A. LDs formation was induced by Oleic acid in HepG2 transfected with GFP-Rab40c for 0, 12, 24 and 48 h, respectively, and LDs were revealed with Oil red. immuno-fluorescence microscopy showed that GFP-Rab40c is recruited to LDs upon stimulation in a time course dependent manner. B. HepG2 cells were co-transfected with pCIneo-Rab40c and GFP-ERGIC-53, then starved for 24 h (upper panels) or stimulated with Oleic acid for 24 h afterward(lower panels), the expressed Rab40c was revealed by immuno-staining with antibody against Rab40c. The data showed that most of the expressed Rab40c co-localized with ERGIC-53 under starvation condition. However, when stimulated with oleic acids, the expressed Rab40c segregated from ERGIC-53, and become associated with the enlarged vesicles characteristic of LDs (indicated by arrow heads). Bar, 20 µm.

Since Rab40c associates with LDs, we examined whether Rab40c is involved in LDs biogenesis. Because it is difficult to transfect GFP-Rab40c into 3T3-L1 adipocytes, we investigated the dynamic membrane association of GFP-Rab40c during LDs formation in HepG2 cells induced with oleic acids, mimicking the the formation of LDs during adipocyte differentiation. It was observed through immuno-fluorescence microscopy that GFP-Rab40c is recruited to the surface of LDs upon stimulation for the formation of LDs in a time course dependent manner. As shown in Figure 4A, Rab40c was associated with LDs after 24 h stimulation, although the majority of Rab40c is located at the position close to LDs. Most of GFP-Rab40c is seen to locate to the surface of LDs after 48 h stimulation.

As the biogenesis of LDs is closely related to the ER, we examined whether Rab40c is also associated with the ER by co-expressing pCIneo-Rab40c and mCherry-Sec61, and found Rab40c did not co-localize with Sec61 (data not shown). However, a pool of Rab40c was seen to associate with ERGIC-53 containing compartments (Figure 3E). Interestingly, most of the expressed Rab40c is present in the area adjacent to ERGIC-53 labeled structures in HepG2 cells under serum starvation condition (Figure 4B, upper panels), but when stimulated with oleic acids, the expressed Rab40c was seen to gradually segregate from ERGIC-53, and increasingly being associated with the enlarged vesicles characteristic of LDs (Figure 4B, lower panels).ERGIC-53 is the marker for ER-Golgi intermediate compartment (ERGIC), possessing ER retention signal and mediating cargo transport from the ER exit-sites, and dynamically recycling between the ER and Golgi apparatus. This result revealed that there exists a dynamic distribution of Rab40c between the ERGIC and LDs, indicating Rab40c is likely involved in the biogenesis of LDs from the ER or ER-derived compartments such as ERGIC.

**Figure 5:**
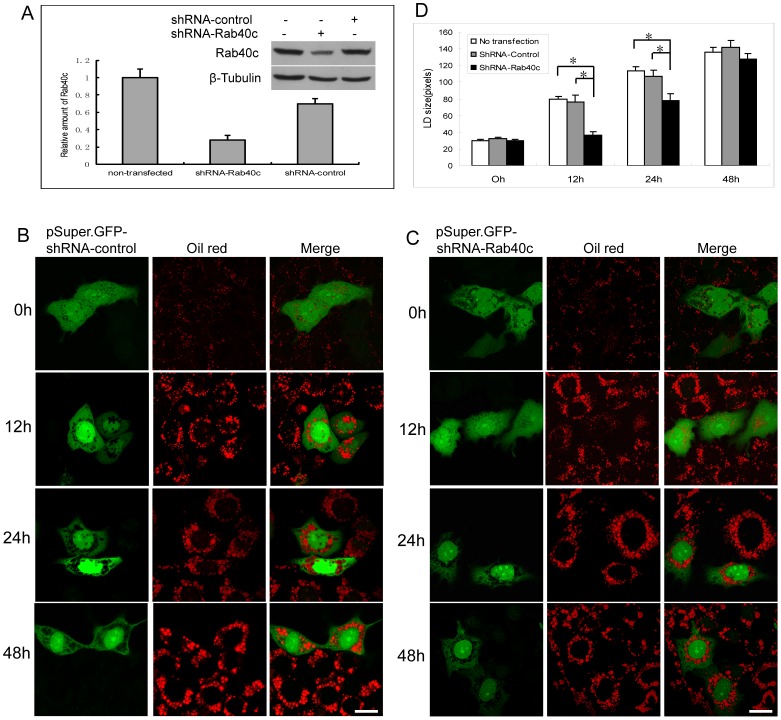
The effects of Rab40c knockdown on lipid droplets formation. A. HepG2 cells were transfected with shRNA-Rab40c or shRNA-control to achieve about 70% transfection efficiency. Western-blot showed that the protein level of Rab40c can be specifically knocked down by shRNA-Rab40c in HepG2 cells. Quantitative analysis was done by densitometry, and the data represent the mean value from 3 independent experiments. B. HepG2 cells were transfected with pSuper.GFP-shRNA-control, then stimulated with oleic acid for the indicated time, and LDs were revealed by Oil red, the data showed that shRNA-control has little effects on LDs. C. HepG2 cells transfected with pSuper.GFP-shRNA-Rab40c, and processed for immuno-fluorescence microscopy analysis as mentioned above, the results revealed that shRNA-Rab40c moderately decreased the size of LDs. D. Quantitative analysis of the mean size of LDs from 50 transfected cells, showing depletion of Rab40c significantly decreases the size of LD. *p<0.05. The error bars represent the deviation of three independent experiments. Bar, 20 µm.

To further investigate the role of Rab40c in LDs biogenesis, we performed RNAi experiments to see whether the depletion of Rab40c affects the formation of LDs. HepG2 cells were transfected with pSuper-shRNARab40c, then stimulated with oleic acids, and the formation of LDs was analysed with confocal microscopy. The knockdown efficiency was monitored by western-blot using antibody against hRab40c (Figure 5A). It was observed that the control knockdown has little effects on the formation of LDs (Figure 5B). However, depletion of Rab40c with shRNA-Rab40c moderately affected the size of LDs (Figure 5C). The average size of LDs of 50 Rab40c-knockdown cells was further analysed with imageJ software compared with control knockdown cells, and the mean size of lipid droplet was further analyzed with *q test* statistical method. The results indicate that depletion of Rab40c significantly decreases the size of LDs (Figure 5D). In addition, this effect was more obvious upon stimulation for 12 h and 24 h, suggesting Rab40c may play a role during the early stages of LDs biogenesis.

### Rab40c is Potentially Involved in Adipocyte Differentiation and Interacts with TIP47

**Figure 6:**
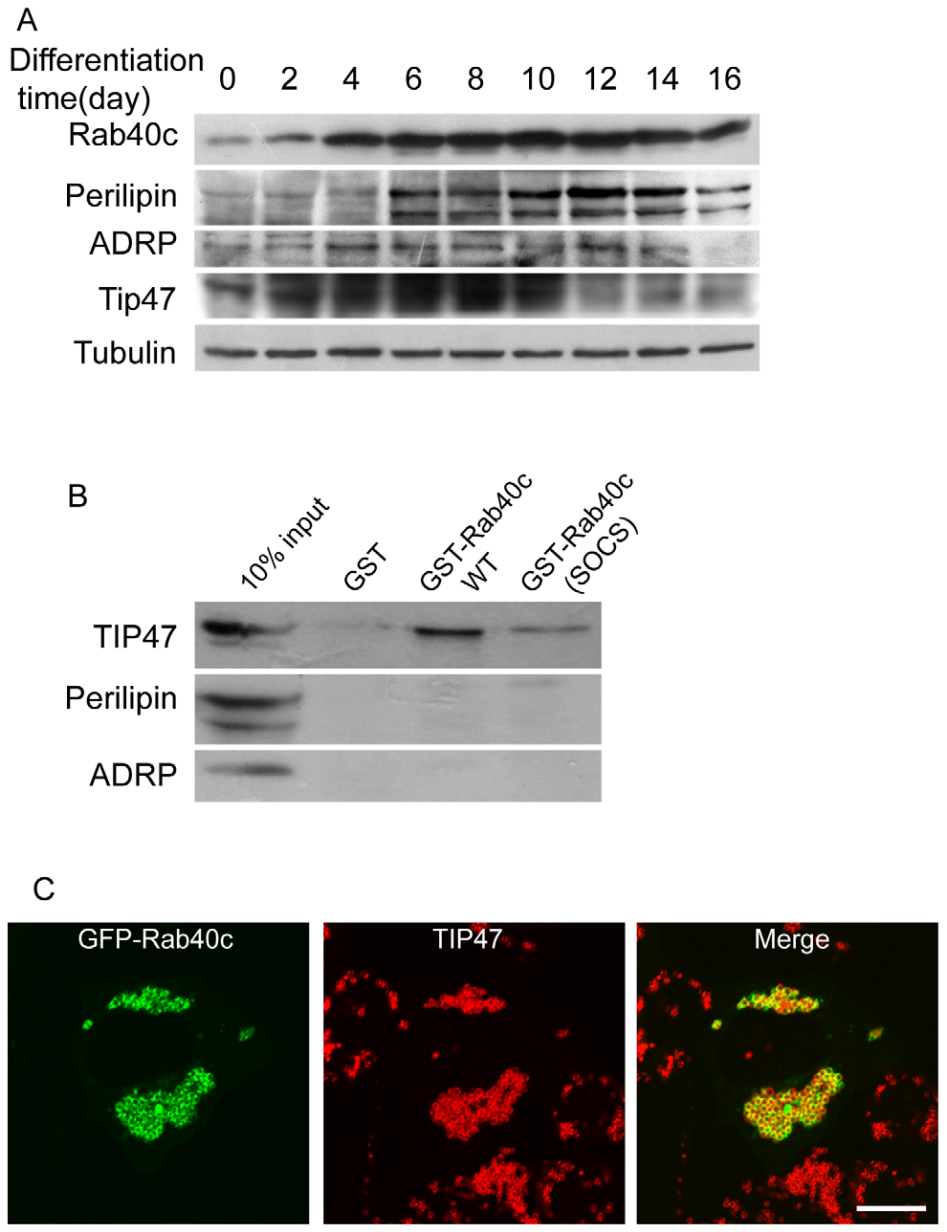
Rab40c is involved in adipocyte differentiation and interacts with TIP47. A. 3T3-L1 cells were induced to differentiate into adipocytes for the indicated times, then the cells were lysed and subjected for western-blot to detect β-tubulin, Rab40c, Perilipin, ADRP or TIP47. The results demonstrated that the level of Rab40c increased during adipocyte differentiation. B. 3-T3-L1 cell lysates were incubated with GST, GST-Rab40cWT or GST-Rab40c(SOCS) coupled to GST-Sepharose 4B resin. Proteins retained on the beads were processed for western-blot using antibodies against TIP47, Perilipin or ADRP. The data demonstrated that Rab40c can interact with TIP47 but not ADRP and Perilipin. Mutations of SOCS box in Rab40c disrupted the interaction between Rab40c and TIP47. C. Immuno-fluorescence microscopy revealed that GFP-Rab40c exhibites close apposition with TIP47 upon oleic acids stimulation in HepG2 cells. Bar, 20 µm.

The above data demonstrated that Rab40c is likely involved in the biogenesis of LDs, so we examined the expression of Rab40c during adipocyte differentiation to see whether Rab40c is involved in adipocyte differentiation. 3T3-L1 cells were induced to differentiate into adipocytes, and the expression of Rab40c was examined using peptide antibody against Rab40c. The results showed that the level of Rab40c protein increases following with the adipocyte differentiation (Figure 6A), suggesting that Rab40c may potentially play a role in adipocyte differentiation. The protein levels of ADRP, Perilipin and TIP47 were also examined, and the data were consistent with the previous studies [23].

As PAT family proteins play key roles in the biogenesis of lipid droplets, we detected the interaction between Rab40c and ADRP, Perilipin or TIP47 through GST-pulldown assay. Interestingly, we found that Rab40c can interact with TIP47, but not ADRP and Perilipin (Figure 6B). Furthermore, mutation of SOCS box in Rab40c disrupts the interaction between Rab40c and TIP47. Immuno-fluorescence microscopy also revealed that GFP-Rab40c exhibites close apposition with TIP47 upon oleic acids stimulation in HepG2 cells (Figure 6C).

### Over-expression of Rab40c Induces the Clustering of LDs

**Figure 7:**
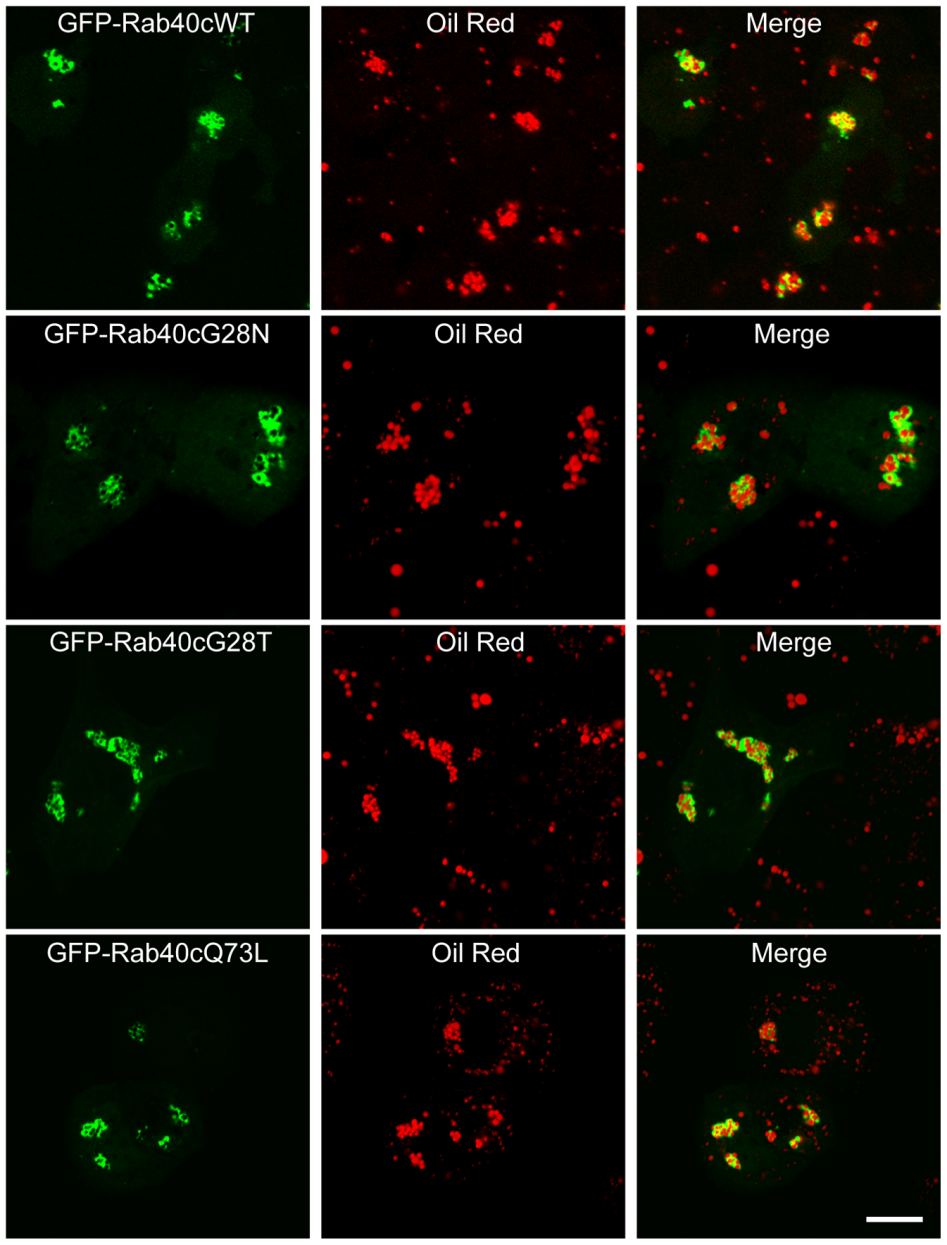
Expression of Rab40c induces the clustering of LDs. NRK cells were transfected with GFP-Rab40cWT (wild type), GFP-Rab40cG28N, GFP-Rab40cG28T or GFP-Rab40cQ73L, respectively. Then the cells were labeled with Oil red and processed for confocal microscopy analysis. The results demonstrated that GFP-Rab40cWT and all the mutants can significantly induce the clustering of LDs. Bar, 20 µm.

Upon over-expression, Rab40c was mostly observed in the clustered LDs. Over-expression of GFP-Rab40cWT (wild type) significantly resulted in the clustering of oil red labeled LDs in NRK cells (Figure 7, upper panels). As indicated in Figure 1A, the conserved PM1 motif in Rab40c is GxxxGKG, so we converted residue G to N or T to generate the expression construct GFP-Rab40cG28N or GFP-Rab40cG28T, which usually represents the negative form or wild type form for other Rab proteins, as well as the active form GFP-Rab40cQ73L. Next we examined whether these alternations in Rab40c will affect its effects on the distribution of LDs. The results demonstrated that all these Rab40c mutants can significantly induce the clustering of LDs (Figure 7), meaning the effect of Rab40c on inducing the clustering of LDs is likely independent of its GTPase activity. In addition, it was observed that over-expression of non-tagged Rab40c also induces the clustering of LDs (Figure 3C), excluding that this effect was caused by GFP tag.

### SOCS Box in Rab40c is Essential for Inducing the Clustering of LDs

**Figure 8:**
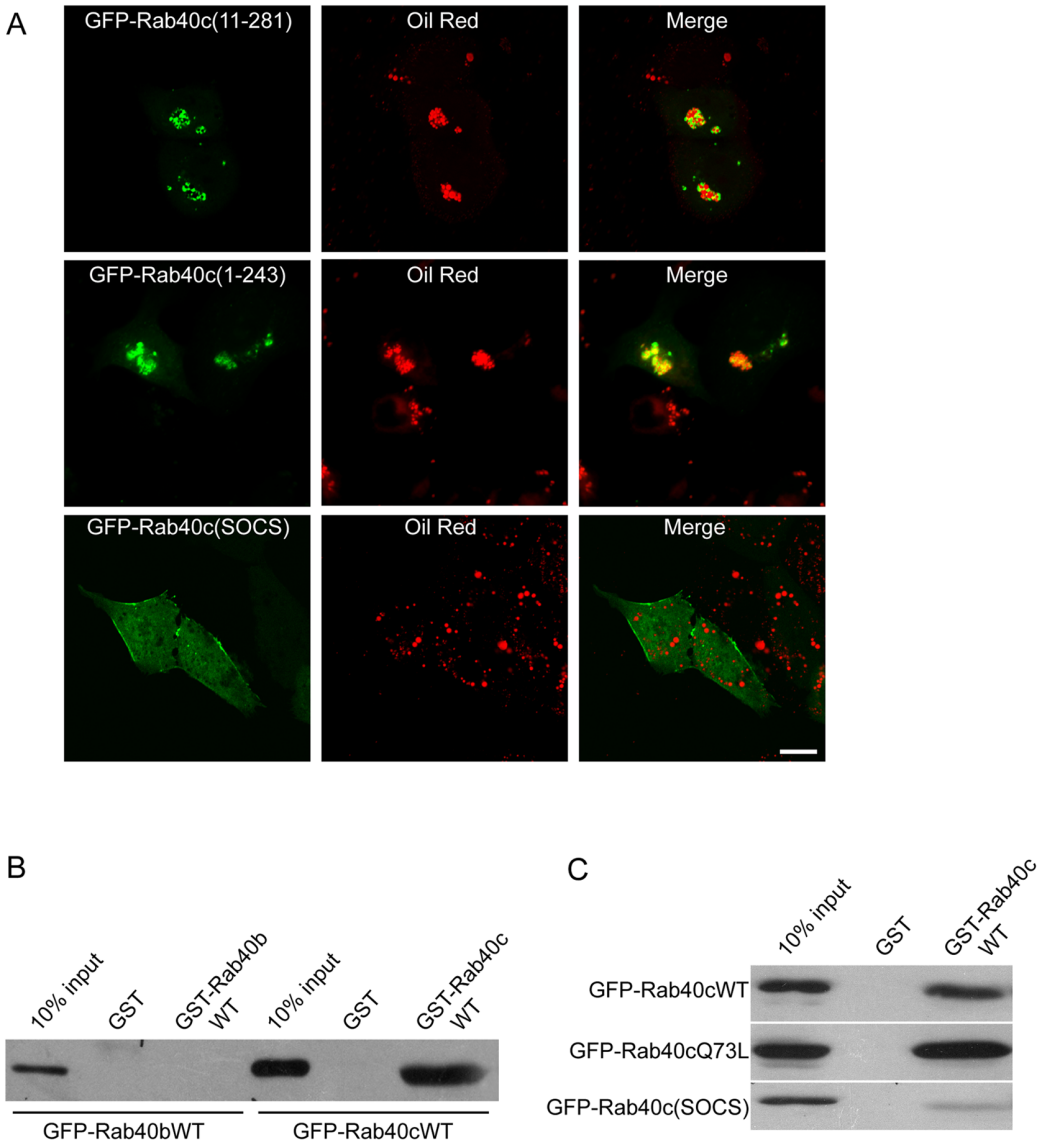
SOCS box in Rab40c mediates self-interaction and is essential for inducing the clustering of LDs. A. NRK cells were transfected with the N-terminal truncated form GFP-Rab40c(11-281), C-terminal truncated form GFP-Rab40c(1-243) or GFP-Rab40c(SOCS) (mutant with mutations LPLP(212-215)AAAA in SOCS box), respectively. The cells were labeled with Oil red and processed for immuo-fulorescence microscopy. The results revealed that disruption of the SOCS box abolished the association of Rab40c with lipid droplets, and its ability to induce the clustering of LDs. B. MCF7 cell lysates expressing GFP-Rab40b or GFP-Rab40c were subjected to pull-down experiments using immobilized GST-Rab40b or GST-Rab40c. Western-blot experiments using GFP antibody demonstrated that GST-Rab40c can efficiently retain GFP-Rab40c, where as GST-Rab40b did not exhibit binding to GFP-Rab40b. C. MCF7 cell lysates expressing GFP-Rab40cWT, GFP-Rab40cQ73L or GFP-Rab40c(SOCS) were subjected to pull-down experiments using GST-Rab40c, showing that GST-Rab40c can bind to both GFP-Rab40c and GFP-Rab40cQ73L, but not GFP-Rab40c(SOCS) mutant. Bar, 20 µm.

To characterize the structural sequences responsible for Rab40c in inducing the clustering of LDs, we expressed the N-terminally truncated form GFP-Rab40c (11-281) and C-terminally truncated form GFP-Rab40c (1-243) in NRK cells, and found that both the N-terminal variable region and C-terminal variable region are not essential for inducing the clustering of LDs (Figure 8A). However, disruption of the function of SOCS box by mutations LPLP(212-215)AAAA abolished the association of Rab40c with LDs. furthermore, the LDs did not cluster, but are dispersed in the cytoplasm (Figure 8A, lower panels), suggesting that the SOCS box of Rab40c is essential for its ability to associate with LDs and to induce the clustering of LDs.

Further investigation demonstrated that Rab40c may form homodimer or homooligomer through SOCS box. To test the self-interaction of Rab40c, GST-Rab40c coupled to GST sepharose beads was used to bind GFP-Rab40c in MCF7 cell lysates. As shown in Figure 8B, GST-Rab40c but not GST can efficiently bind to GFP-Rab40c. Under similar condition, GST-Rab40b did not exhibit self-interaction, suggesting that Rab40c specifically interacts with itself. Mutations in SOCS box abolished the self-interaction of Rab40c (Figure 8C), as well as the interaction between Rab40c and TIP47 (has been described in Figure 6B). These results indicated that the self-interaction through SOCS box plays an important role in the interaction with TIP47, association with LDs and inducing the clustering of LDs.

## Discussion

Proteomics studies revealed that many Rab GTPases are likely associated with LDs, but the members of the associated Rab proteins may vary in different experiments or cell types [26]–[28], suggesting that different Rab proteins will be recruited onto the LDs under different conditions, giving the reason that Rab40c was not previously reported to be found on LDs.

Rab40c was shown to play roles in gastrulation of Xenopus embryo and in vesicle transport in oligodendrocytes [32], [33]. However, the cell biological functions of Rab40c in mammalian cells have not elucidated in detail, and the sub-cellular location of Rab40c remains controversy. In this study, we presented our results showing that Rab40c is a novel LDs associated Rab protein, and Rab40c knockdown slightly decreases the size of LDs. Since Rab proteins are the key regulators for membrane trafficking, Rab40c may play a role in regulating membrane fusion/fission of LDs through interaction with other factors, which regulating the growth and size of LDs during the maturation of LDs. However, this hypothesis needs further investigation, although it is consistent with the ability of Rab40c to associate LDs and induce clustering of LDs.

The well known factors regulating the biogenesis dynamics of LDs are the PAT family proteins. Perilipin is only expressed in adipocytes and steroidogenic cells, and regulates the formation and lipolysis of LDs [13], [18]. The perilipin knockout mice did no exhibit diet-induced obesity [37]. ADRP and TIP47 are expressed ubiquitously. ADRP is increased in the early stage of adipocyte differentiation and regulates lipid storage in LDs [19], [22], while TIP47 functions in the early stage of adipogenesis and its level decreases with the LDs maturation [23]. The underlying mechanisms for Rab40c association with LDs and potential role in regulating the biogenesis of LDs were investigated in this study. Our results indicate that Rab40c interacts with PAT protein TIP47, which has also been shown as an effector of Rab9 regulating M6PR trafficking [38]–[40]. Like TIP47, Rab40c might also play a role in the early stage of LDs biogenesis, so the interaction between Rab40c and TIP47 may be crucial for Rab40c in associating with LDs, and modulating their biogenesis.

LDs is most likely derived from the ER or its associated membrane [11]–[13]. However, in this study, we observed that a fraction of Rab40c is associated with the membrane structures marked by ERGIC-53 especially under serum starvation. ERGIC-53 is a marker for ERGIC., Interestingly, Rab40c was seem to be increasingly recruited to LDs during LDs formation. It was reported that over-expressing Rab18 induces apposition of LDs to ER membrane cisternae, but ER location of Rab18 was not observed [30]. Since ERGIC-53 possesses ER retention signal and mediating cargo transport from ER exit-sites, and dynamically recycling between the ER and Golgi apparatus [41], [42], and a recent finding indicates that ERGIC-53 distributes from the ER to Golgi depending on physiological condition [43]. Therefore, we hypothesized that Rab40c may be involved in the process of LDs biogenesis from the ER or ER-derived membrane compartments such as ERGIC. More experiments are needed to explore this possibility and to investigate whether ERGIC is involved in LDs formation.

Rab40c may regulate the distribution of LDs, as over-expressing Rab40c caused the clustering of LDs, this effect depends on the intact SOCS box. The SOCS (suppressors of cytokine signaling) box is a structural domain found at the C-terminus of over 70 human proteins. The SOCS box can interact with Cullin−/ElongB/C to form E3 ligase complexes, mediating cytokine receptors for proteasomal degradation [44]. In this study, we found that Rab40c interacts with itself through SOCS box, in addition, SOCS box is also responsible for Rab40c in interacting with TIP47, indicating that the formation of homo-dimers and interaction with TIP47 through SOCS box is essential for Rab40c to associate with LDs and in inducing the clustering of LDs, providing a novel mechanism for SOCS box containing proteins.
